# A huge uterine leiomyoma in a 15-year-old girl

**DOI:** 10.1093/omcr/omad058

**Published:** 2023-06-26

**Authors:** Abdelhamid Benlghazi, Moad Belouad, Saad Benali, Moulay A B Habib, Moulay M E Hassani, Jaouad Kouach

**Affiliations:** Department of Gynecology and Obstetrics, Military Hospital of Instruction Mohamed V Rabat, Rabat, Morocco; Department of Gynecology and Obstetrics, Military Hospital of Instruction Mohamed V Rabat, Rabat, Morocco; Department of Gynecology and Obstetrics, Military Hospital of Instruction Mohamed V Rabat, Rabat, Morocco; Department of Gynecology and Obstetrics, Military Hospital of Instruction Mohamed V Rabat, Rabat, Morocco; Department of Gynecology and Obstetrics, Military Hospital of Instruction Mohamed V Rabat, Rabat, Morocco; Department of Gynecology and Obstetrics, Military Hospital of Instruction Mohamed V Rabat, Rabat, Morocco

Leiomyomas are noncancerous tumors that frequently develop in the uterus of adult women, with a prevalence of 20–30% among women aged 35–50. These tumors are rare in women younger than 15 years old, and only a few cases of symptomatic uterine leiomyomas have been documented in adolescents, as reported in the literature [1]. Typically, leiomyomas are discovered incidentally during imaging examinations, and they may cause abdominal pain or abnormal vaginal bleeding [2].

A 15-year-old girl with no previous medical history came to our department complaining of severe abdominal and pelvic pain and abdominal distension. On physical examination, her abdomen was distended with the presence of a huge abdominal and pelvic mass on palpation.

A pelvic ultrasound showed a large round mass, slightly heterogeneous, within the uterus, measuring 21 × 20 cm, and highly vascularized on color Doppler. An abdominal-pelvic computed tomography also showed the presence of a large heterogeneous round mass measuring 22 × 19 cm in the lower abdomen and pelvis, located above the uterus.

**Figure 1:**
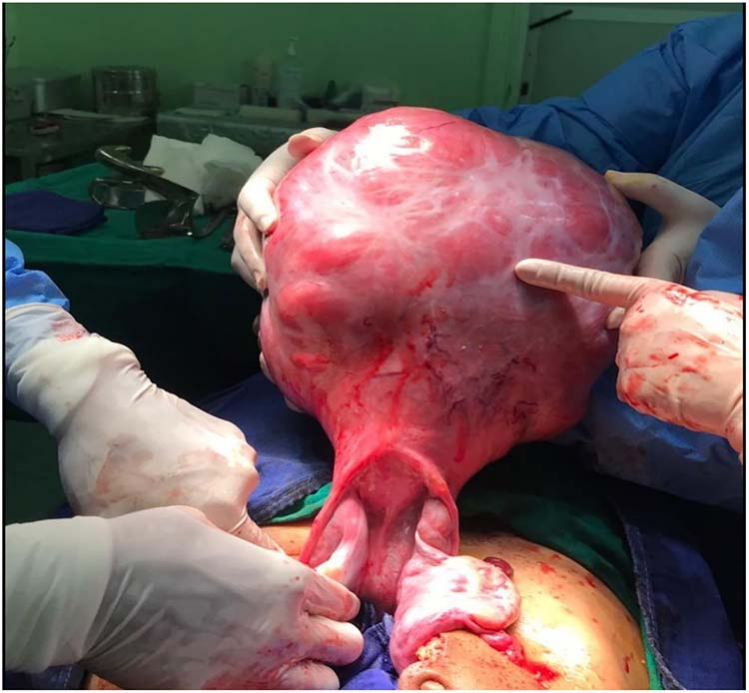
Intraoperative image of the exteriorized uterus, showing the huge fundal leiomyoma.

During operative exploration, a large mass was found in the uterine fundus measuring 25 × 20 cm (see Fig. 1). The ovaries and fallopian tubes were normal. An open myomectomy was performed and the histological findings were in favor of a uterine myoma. The patient had an uncomplicated postoperative course.

It is crucial to keep in mind that leiomyomas should be considered as a significant differential diagnosis among adolescents. Myomectomy represents a beneficial option to preserve the fertility function of adolescents who suffer from leiomyomas.
